# Investigation on preparation and performance of spinel LiNi_0.5_Mn_1.5_O_4_ with different microstructures for lithium-ion batteries

**DOI:** 10.1038/srep13299

**Published:** 2015-08-24

**Authors:** Yuan Xue, Zhenbo Wang, Lili Zheng, Fuda Yu, Baosheng Liu, Yin Zhang, Ke Ke

**Affiliations:** 1 School of Chemical Engineering and Technology, Harbin Institute of Technology, No. 92 West-Da Zhi Street, Harbin, 150001 China; 2 Chilwee Power Co. Ltd., No. 12 Zhizhou Road, Xinxing Industrial Park, Zhicheng, Changxing, Zhejiang Province 313100, China

## Abstract

The high voltage spinel LiNi_0.5_Mn_1.5_O_4_ is a promising cathode material in next generation of lithium ion batteries. In this study, LiNi_0.5_Mn_1.5_O_4_ with various particle microstructures are prepared by controlling the microstructures of precursors. LiNi_0.5_Mn_1.5_O_4_ spinel samples with solid, hollow and hierarchical microstructures are prepared with solid MnCO_3_, hollow MnO_2_ and hierarchical Mn_2_O_3_ as precursor, respectively. The homemade spinel materials are investigated and the results show that the content of Mn^3+^ and impurity phase differ much in these three spinel samples obtained under the same calcining and annealing conditions. It is revealed for the first time that an inhomogeneous migration of atoms may introduce Mn^3+^ and impurity phase in the spinel. The hierarchical microstructure with the primary particles interconnected is optimal for electrode materials because this microstructure has a higher conductivity between the interconnected primary particles and appropriate specific surface area. LiNi_0.5_Mn_1.5_O_4_ in this microstructure has the best rate capability and also the best long-term cycling stability.

With an escalating energy crisis and greenhouse gas emission issue, clean energy sources and electric vehicles (EVs) have increasingly attracted much attention. For an effective utilization of clean energy, efficient energy storage systems (ESSs) are indispensible. Among the well-developed ESSs, rechargeable lithium-ion batteries (LIBs) dominate in portable devices such as cell phones, laptops and etc. and are expected to dominate EVs in the near future. In the application of LIBs in EVs, however, it remains a challenge to increase the energy density and power density of the electrode active materials. As a positive electrode active materials for LIBs, LiNi_0.5_Mn_1.5_O_4_ has been widely investigated for its high operation voltage (high energy density) and facile three dimensional lithium-ion diffusion (high power)^1^,^2^,^3^,^4^,^5^.

Based on a mechanism analogous to the Kirkendall effect, a hollow structured LiNi_0.5_Mn_1.5_O_4_ has been synthesized by a facile impregnation approach^6^. The hollow structured LiNi_0.5_Mn_1.5_O_4_ exhibits superior electrochemical performance. On the other hand, hollow manganese oxides microstructures can be synthesized by a simple method from MnCO_3_^7^,^8^. Here, we present a strategy to controllably synthesize LiNi_0.5_Mn_1.5_O_4_ with various microstructures by using manganese compounds with different microstructures. The effects of microstructures on performance of LiNi_0.5_Mn_1.5_O_4_ were investigated systematically.

LiNi_0.5_Mn_1.5_O_4_ containing only Mn^4+^ has a poor electronic conductivity because active Ni^2+/3+/4+^ redox centers are isolated among the inactive Mn^4+^ nearest neighbors^9^,^10^. LiNi_0.5_Mn_1.5_O_4_ spinels with presence of Mn^3+^ exhibit better rate performance due to a higher electronic and lithium-ion conductivity^11^,^12^. However, Mn^3+^ ions are unstable and tend to undergo a disproportionation reaction: 2Mn^3+^ = Mn^2+^ + Mn^4+^. The produced Mn^2+^ ions then dissolve into the electrolyte, leading to a capacity fading during charging-discharging cycling^13^,^14^. In the LiNi_0.5_Mn_1.5_O_4_ products, Li_x_Ni_1-x_O usually appears as impurity phase, lowering the capacity and blocking Li^+^ mobility in the material. In other words, Mn^3+^ and impurity in the spinel have great effects on the electrochemical properties. Therefore, it is crucial to find out the reasons of forming Mn^3+^ and impurity in the spinel product and control their content^15^,^16^,^17^. Loss of oxygen occurring at high-temperature calcination will obviously lead to the appearance of impurity phase and Mn^3+^ in the spinel. The oxygen loss is reversible and can be recovered by annealing, which can also reduce impurity and Mn^3+^ content^18^,^19^. Besides, Mn^3+^ and impurity content in the spinel is affected by a cation doping^20^,^21^. Here, for the first time, we have revealed that an inhomogeneous atoms migration may also introduce Mn^3+^ and impurity phase in the spinel.

## Experimental

### Preparation of precursors with different microstructures

Firstly, solid MnCO_3_ spheres were synthesized by a precipitation method described as below. 200 mL ethanol was added to a MnSO_4_ solution (0.04 mol L^−1^, 1 L) to form a mixture. A NH_4_HCO_3_ solution (0.4 mol L^−1^, 1 L) was added to the mixture solution dropwisely under stirring. The mixture was kept stirring for 5 h. Then the produced precipitate was collected and dried at 60 °C to obtain solid spherical MnCO_3_.

The obtained MnCO_3_ was treated by different methods to change the microstructure. MnO_2_ with a hollow microstructure was synthesized by calcinating the solid MnCO_3_ spheres at 350 °C for 4 h with a subsequent acid-washing. 0.1 mol L^−1^ HCl solution was employed in the acid-washing process. Mn_2_O_3_ with a hierarchical microstructure was synthesized by calcinating the solid MnCO_3_ spheres at 800 °C for 4 h.

### Preparation of LiNi_0.5_Mn_1.5_O_4_ with different microstructures

LiNi_0.5_Mn_1.5_O_4_ spinels with various microstructures were prepared by using the above-mentioned solid MnCO_3_ spheres, hollow MnO_2_ and hierarchical Mn_2_O_3_ as precursors respectively. Stoichiometric amounts of LiOH·H_2_O, Ni(NO_3_)_2_·6H_2_O and the corresponding precursor taken in the ratio Li:Ni:Mn = 1.05:0.5:1.5 were dispersed in ethanol. The dispersion was stirred to mix the reactants and the ethanol was evaporated slowly at room temperature under stirring. The mixture obtained after evaporation was calcined at 850 °C for 10 hours and then cooled at a rate of 0.5 °C min^−1^ in the air to obtain the product. The product was solid LiNi_0.5_Mn_1.5_O_4_ spheres (denoted as SO-LNMO), hollow LiNi_0.5_Mn_1.5_O_4_ (denoted as HO-LNMO) and hierarchical LiNi_0.5_Mn_1.5_O_4_ (denoted as HI-LNMO), corresponding to the precursor of solid MnCO_3_ spheres, hollow MnO_2_ and hierarchical Mn_2_O_3_, respectively.

### Characterization

The obtained materials were characterized by scanning electron microscopy (SEM), X-ray photoelectron spectroscopy (XPS) and powder X-ray diffraction (XRD). XRD characterization was carried out with a D/max-RB diffractometer using a Cu Kα source and recorded with a step of 0.05°. XPS characterization was carried out with Thermofisher Scienticfic, K-Alpha. Brunauer–Emmett–Teller (BET) specific surface area was tested by using Beishide 3H-2000Ps1. And the Raman spectra were obtained with a Renishaw in Via Raman microscope. The synthesized samples were analyzed by inductively coupled plasma (ICP, PerkinElmer, Optima 5300DV) tests.

Electrochemical tests of the homemade samples were carried out by using coin-type cells (2025). The cathode materials of the cells were made from a slurry containing 80 wt.% active material, 10 wt.% conductive acetylene black as conductive agent and 10 wt.% polyvinylidene fluoride (PVDF) as binder dissolved in n-methyl pyrrolidinone. The slurry was evenly coated onto an aluminum foil by using a blade and then dried in a vacuum oven at 120 °C for overnight. Then the foil was punched into a circular electrode (1.4 cm in diameter). The loading weight of the active materials on the electrode was about 2 mg cm^-2^. Cells with lithium metal as the counter electrode were assembled in an argon-filled glove box. The electrolyte was 1 mol L^−1^ LiPF_6_ in a mixture of ethylene carbonate and dimethyl carbonate with a ratio of 1:1 by weight. Charge-discharge tests were carried out on a NEWWARE battery tester. When the current densities were higher than 0.5 C, the cells were charged galvanostatically to 4.95 V and the cell voltage was kept at 4.95 V until the current decreased to 0.1 C. Then the cells were discharged to 3.5 V at different rates. Cyclic voltammetry (CV: 3.5–5.1 V, 0.1 mV s^-1^) and electrochemical impendence spectroscope (EIS) was carried out on a CHI650D electrochemical workstation. EIS measurements were conducted with an AC amplitude of 5 mV at 4.73 V in the frequency range from 10^5^ Hz to 0.01 Hz.

## Results and Discussion

The procedure of microstructure-controllably preparing LiNi_0.5_Mn_1.5_O_4_ is illustrated in Figure S1 in Supporting Information. As is shown, the microstructures of LiNi_0.5_Mn_1.5_O_4_ can be controlled by using manganese compound with different microstructures as precursor.

### Precursors with different microstructures obtained at different conditions

**Figure 1:**
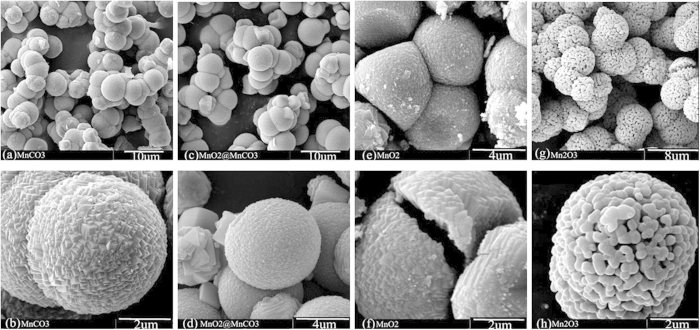
SEM micrographs of materials obtained during the procedure of synthesizing precursors: (**a**,**b**) MnCO_3_ precipitate before calcination, (**c**,**d**) MnO_2_/MnCO_3_ composite obtained after calcinating the precipitate at 350 °C, (**e**,**f**) MnO_2_ obtained after acid-washing the MnO_2_/MnCO_3_ mixture and (**g**,**h**) Mn_2_O_3_ obtained after calcinating the MnCO_3_ precipitate at 800 °C.

The produced precursors were characterized by XRD and SEM, as shown in Figure S2 and Fig. 1.

Solid spheres, the precipitate before calcination, shown in Fig. 1(a,b) are synthesized by a precipitation reaction between MnSO_4_ and NH_4_HCO_3_. The XRD pattern (Figure S2(a)) of the obtained precipitate can be assigned to MnCO_3_. The MnCO_3_ precipitate appears to be solid microspheres with a diameter of ~5 um.

After the MnCO_3_ spheres was calcined at 350 °C for 4 h, the XRD pattern in Figure S2(b) indicates that the MnCO_3_ precipitate turns into a mixture of MnCO_3_ and MnO_2_. The MnCO_3_ precipitate was partially decomposed to MnO_2_ whereas the inner core remains to be MnCO_3_^7^. MnCO_3_ can be easily dissolved in a diluted acid, whereas MnO_2_ cannot. Therefore, after acid-washing the partially decomposed samples, the MnCO_3_ residual almost disappeared as indicated by a weakened MnCO_3_ peaks in the XRD pattern in Figure S2(c) and the MnO_2_ peaks remains without obvious change. In addition, a weight loss of the sample after acid-washing also supports the dissolution of the undecomposed MnCO_3_. The morphology of the sample after acid-washing is shown in Fig. 1(e,f). The particles have spherical shape with a hollow microstructure seen from the cracked particle.

After the precipitate MnCO_3_ spheres was calcined at 800 °C for 4 h, it turns into Mn_2_O_3_ as indicated by the XRD pattern shown in Figure S2(d). Different from the calcinations at 350 °C, the microstructure of the produced Mn_2_O_3_ particles changed into a submicro/micro hierarchical structure after calcination at 800 °C, as shown in Fig. 1(g,h). The primary particle size of the Mn_2_O_3_ is about 400 nm and the secondary particles are microspheres with diameters of ~5 um. It is worth noting that the primary particles are interconnected, rather than closely piled up. It is attributed to that the hierarchical Mn_2_O_3_ microstructure is formed by a volume shrinkage of the MnCO_3_ microsphere occurred at calcinations.

### LiNi_0.5_Mn_1.5_O_4_ with different microstructures produced from different precursors

**Figure 2:**
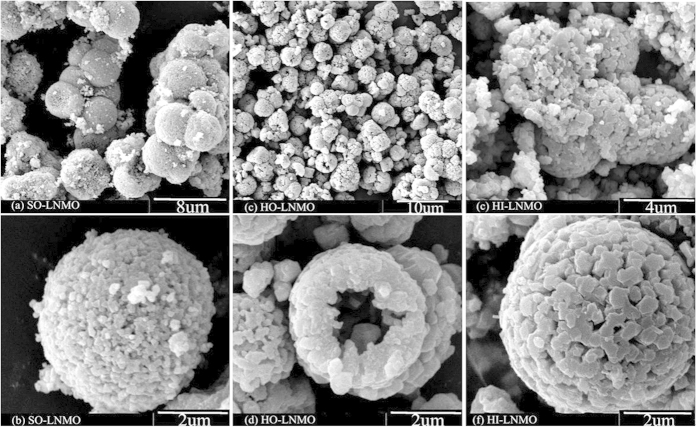
SEM micrographs of (**a**,**b**) SO-LNMO prepared from a solid-structured precursor, (**c**,**d**) HO-LNMO prepared from a hollow-structured precursor and (**e**,**f**) HI-LNMO prepared from a hierarchical-structured precursor.

The SO-LNMO, HO-LNMO and HI-LNMO are synthesized by using the above-mentioned solid MnCO_3_ spheres, hollow MnO_2_ and hierarchical Mn_2_O_3_ as precursors, respectively. Figure 2 shows SEM images of these produced LiNi_0.5_Mn_1.5_O_4_ materials. All the three samples remain the spherical shape of the precursors.

As shown in Fig. 2(a,b), the SO-LNMO particle prepared with the solid-sphere-structured precursor are solid seen from the section of particles. As shown in Fig. 2(c,d), the HO-LNMO particle prepared with the hollow-sphere-structured precursor has a similar hollow microstructure with a dense shell (~1 um in thickness). As shown in Fig. 2(e,f), the HI-LNMO particle prepared with the hierarchical-structured precursor has a similar submicro/micro hierarchical structure with the primary particles connected. The primary particle size is about 500 nm and the secondary particle is in a shape of microsphere with a diameter of ~5 um.

By a comparison of the microstructure between the LiNi_0.5_Mn_1.5_O_4_ products and their precursors, it can be seen that the particle microstructure of the produced LiNi_0.5_Mn_1.5_O_4_ remains almost the same as their precursors’ microstructures. So it is a feasible way to control the microstructures of LiNi_0.5_Mn_1.5_O_4_ by controlling their precursors’ microstructures.

**Figure 3:**
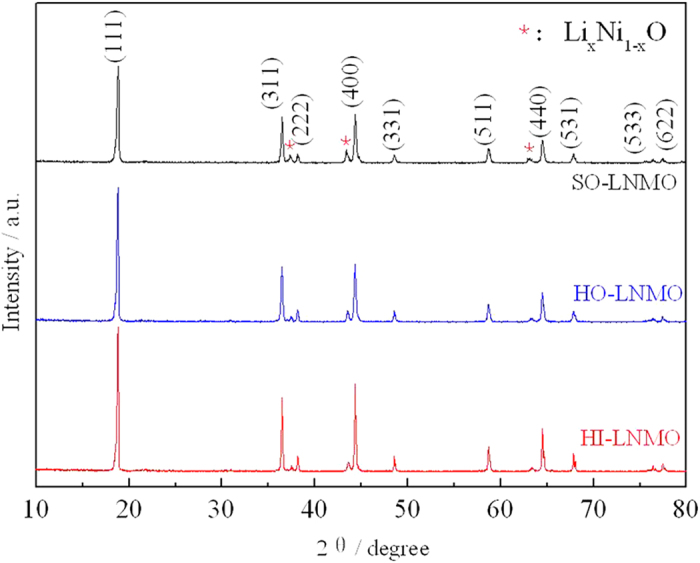
XRD patterns of LiNi_0.5_Mn_1.5_O_4_ with different microstructures.

Figure 3 shows the XRD patterns of the LiNi_0.5_Mn_1.5_O_4_ products with different microstructures. All the three patterns can be assigned to a cubic spinel LiNi_0.5_Mn_1.5_O_4_. Besides, some weak peaks at 2θ = 37.5, 43.6 and 63.4° can be attributed to a Li_x_Ni_1-x_O impurity phase^22^. The content of impurity phase is estimated by calculating the ratio of peak intensity at 2θ = 43.6° of impurity phase to that at 2θ = 44.3° of spinel phase. The intensity ratios in the SO-LNMO, HO-LNMO and HI-LNMO products are 28.1:100, 17.9:100 and 10.4:100, respectively. Impurity content in the three samples decreases in the following order: SO-LNMO > HO-LNMO > HI-LNMO.

With disordered Ni and Mn on the octahedral sites, the cubic spinels have the space group Fd3 m, but the ordering of Ni and Mn in LiNi_0.5_Mn_1.5_O_4_ gives the space group P4_3_32. Raman spectroscopy is a useful tool to determine the cation ordering. Figure S3 shows Raman spectra of the samples. The peaks around 635 cm^−1^ are assigned to the symmetric Mn-O stretching vibration of MnO_6_ group and the peaks around 400 and 490 cm^−1^ can be assigned to the Ni-O stretching mode. The Raman spectra of the three samples are indexed to Fd-3 m space group, due to the absence of a splitting of peaks in the 588–623 cm^−1^ region, which are characteristic of the ordered structure (P4_3_32) of the spinel^23^,^24^.

**Figure 4:**
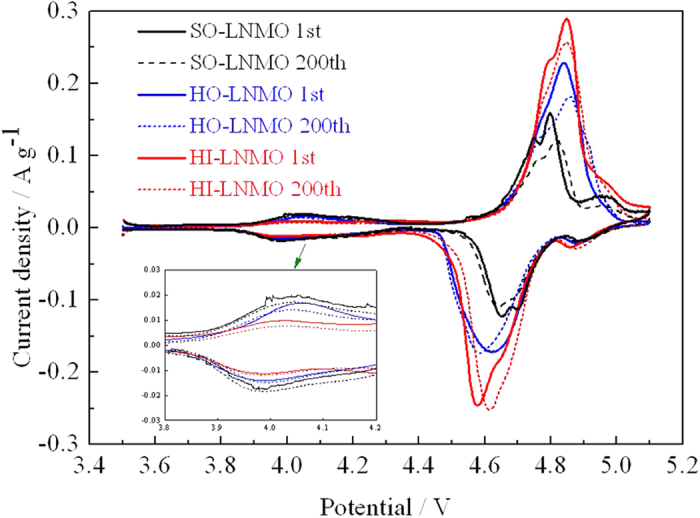
Cyclic voltammetry curves of LiNi_0.5_Mn_1.5_O_4_ with different microstructures before and after 200 cycles at a rate of 2 C.

Cyclic voltammetry (CV) curves of the homemade LiNi_0.5_Mn_1.5_O_4_ with different microstructures before and after 200 cycles at a rate of 2 C are compared (shown in Fig. 4). For all the three samples, the main peaks around 4.7 V are attributed to a Ni^2+^/Ni^4+^redox couple and the weak peaks at 4.0 V are attributed to a Mn^3+^/Mn^4+^ redox couple. Among the three samples, the peak area around 4.7 V of HI-LNMO is the largest, indicating that HI-LNMO has the highest electrolyte-accessible surface area due to its particle microstructure. Obviously, a large specific electrochemically active surface area is beneficial to high rate capability. This will be discussed later. On the other hand, the peaks area at 4.0 V is related to the Mn^3+^ content in spinel^25^. Through comparing the peak area at 4.0 V, the Mn^3+^ content in LiNi_0.5_Mn_1.5_O_4_ is in an order as below: SO-LNMO > HO-LNMO > HI-LNMO.

**Figure 5:**
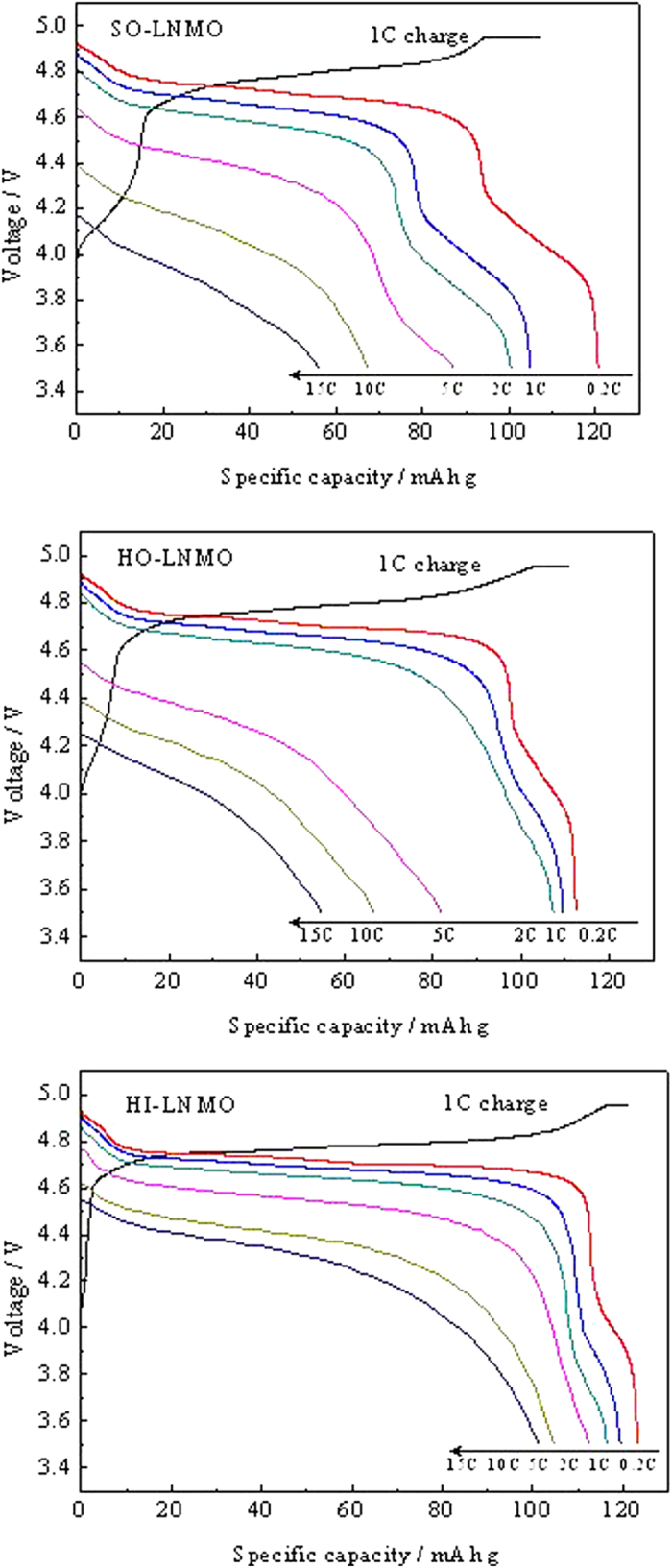
Charge and discharge capacity curves of LiNi_0.5_Mn_1.5_O_4_ with different microstructures.

In order to compare the rate capability among the three LiNi_0.5_Mn_1.5_O_4_ samples, the cells were cycled at various rates ranging from 0.2 to 15 C. The cells were charged at 1 C rate when the discharging current densities were higher than 0.5 C. Figure 5 displays the charging and discharging curves of the three LiNi_0.5_Mn_1.5_O_4_ samples with different microstructures. The curves at low discharging rates show a dominant plateau at around 4.7 V, which is attributed to a Ni^2+^/Ni^4+^ redox couple. A minor plateau in 4 V region is also observed associated with the Mn^3+^/Mn^4+^ redox couple. The length of 4 V plateau in discharging curves can be used to evaluate the relative Mn^3+^ content in the spinel^26^,^27^. The proportion of discharge capacity in 3.8 ~ 4.2 V region at 0.2 C for SO-LNMO, HO-LNMO and HI-LNMO are 17.9%, 10.0% and 7.0%, respectively. This means that Mn^3+^ content in the three samples is in the following order: SO-LNMO > HO-LNMO > HI-LNMO, which is consistent with the estimated results from CV curves.

From above results, it can be seen that the Mn^3+^ and impurity contents in the three samples both are in an order as below: SO-LNMO > HO-LNMO > HI-LNMO. Generally, it is believed that the appearance of Mn^3+^ and impurity phase is caused by a loss of oxygen at high-temperature calcination. So the content of the impurity phase and Mn^3+^ in spinel is affected by calcining and annealing conditions. In this work, however, the calcining and annealing conditions were kept the same for all the three spinel samples. So the difference in the Mn^3+^ and impurity contents among these three samples are rather related to some difference of their precursor particles’ microstructures.

During the synthesis process, precursor, Ni(NO_3_)_2_ and LiOH were dispersed into ethanol to mix these reactants, in which all the Ni(NO_3_)_2_ and a part of LiOH dissolved in ethanol. After the evaporation of ethanol, dissolved Ni(NO_3_)_2_ will deposit on the precursors’ surface. SEM images of the mixture after mixing process are shown in Figure S4. The distribution of the Ni element depends on the precursor particles’ microstructure. The schematic distribution of Ni and Mn elements in the mixture after the evaporation in different methods is shown in Figure S5.

In the case of using solid-sphere-structured precursor, most of the Ni(NO_3_)_2_ deposits on the outer surface of solid MnCO_3_ microspheres as illustrated in Figure S4(a) and Figure S5. During the subsequent high-temperature calcination, Ni and Mn atoms have to migrate over a distance as long as several micrometers to form a homogeneous LiNi_0.5_Mn_1.5_O_4_ phase. This long distance leads to an inhomogeneous elements’ distribution even after calcinations. There will be relatively more Ni element in the surface, resulting in high impurity content and more Mn element in interior, resulting in high Mn^3+^ content of the produced SO-LNMO.

In the case of hollow-structured precursor, Ni(NO_3_)_2_ deposits on both the outer and the inner surfaces of the hollow-sphere’ shell as illustrated in Figure S4(b) and Figure S5. The distance for the migration of Ni and Mn atoms is reduced to at least half of that in the above-mentioned solid-sphere-structured precursor. As a result, the content of Mn^3+^ and impurity phase is expected to be lower than that in the SO-LNMO, which is supported by the actual results of the order in Mn^3+^ and impurity content.

In the case of hierarchical-structured precursor, Ni(NO_3_)_2_ could deposit in submicro-sized space between the primary particles as illustrated in Figure S4(b) and Figure S5. The distance of the migration for Ni and Mn atoms could be reduced to the primary-particle-size level, much shorter than the above-mentioned two cases, leading to an inhomogeneous elements’ distribution. Therefore, HI-LNMO has the lowest Mn^3+^ and impurity contents.

**Figure 6:**
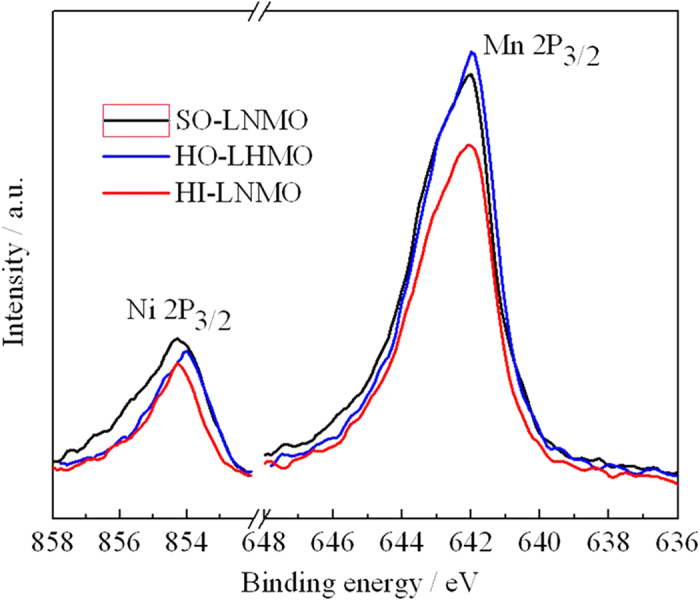
Ni 2p_3/2_ and Mn 2p_3/2_ XPS spectra of LiNi_0.5_Mn_1.5_O_4_ with different microstructures.

To further verify the above discussion about the reason of forming Mn^3+^ and impurity in the spinel, LiNi_0.5_Mn_1.5_O_4_ products after calcinations were characterized by XPS^28^. The Ni 2p_3/2_ and Mn 2p_3/2_ spectra are shown in Fig. 6. The ratio of peak area of Ni 2p_3/2_ to that of Mn 2p_3/2_ in the SO-LNMO, HO-LNMO and HI-LNMO products are 30.9:100, 25.7:100 and 24.5:100, respectively. This result indicates that the Ni content in the surface of SO-LNMO is the highest, which is consistent with the above discussion. The Mn and Ni contents of samples were analyzed by ICP and the results are shown in Table S1. The molar ratios of Mn/Ni in the SO-LNMO, HO-LNMO and HI-LNMO are 3.10, 3.21 and 3.10, respectively. The molar ratios of Mn/Ni in the SO-LNMO and HI-LNMO are same. The molar ratios of Mn/Ni in the samples are inconsistent with the molar ratios of Mn/Ni in the surface. So the difference in distribution of Ni and Mn on the surface come from inhomogeneous migration of atoms, rather than the cation composition in the initial mixtures of reagents.

Besides, the Mn^3+^/Mn^4+^ ratio in the surface was estimated by Mn 2p_3/2_ XPS curve fitting^29^, shown in Figure S6. Mn^4+^ and Mn^3+^ each give rise to the Mn 2p_3/2_ binding energies at 642.9 and 641.9 eV. The ratio of peak area of Mn^4+^ to that of Mn^3+^ in the SO-LNMO, HO-LNMO and HI-LNMO are 39.8:100, 49.4:100 and 49.2:100, respectively. This result indicates that the Mn^3+^ content in the surface of SO-LNMO is the lowest. However, the CV curves and discharging curves have shown that the Mn^3+^ content in SO-LNMO is the highest. It means that the excess Mn^3+^ exists in interior of particles, which is consistent with the above discussion.

**Figure 7:**
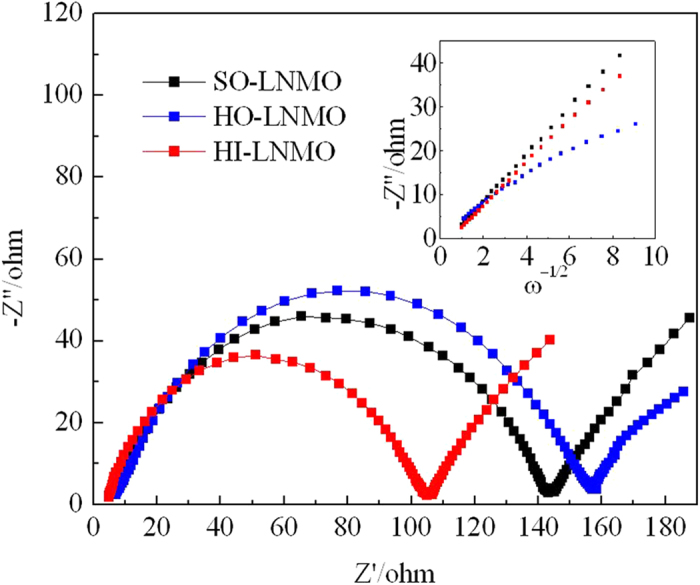
Electrochemical impendence spectroscope of LiNi_0.5_Mn_1.5_O_4_ with different microstructures.

The electrochemical impendence spectroscope measurements were performed on the cells. And the Nyquist plots are shown in Fig. 7. As shown, each plot consists of a depressed semicircle in the high frequency region and a sloping line at low frequency range. The depressed semicircle reflects the interface impendence, including interfacial layer and charge transfer reaction and the sloping line reflects the diffusion of Li-ion in the solid electrode, respectively^30^,^31^. It can be seen that the interface impendence of HI-LNMO is the smallest.

The Warburg coefficient (B) can be obtained from the slope of - Z″ = Bω^−1/2^, showed in inset of Fig. 7. The Warburg coefficient (B) of SO-LNMO, HO-LNMO and HI-LNMO are 5.3, 4.8 and 2.8, respectively. Then the Li-ion diffusion coefficient can be estimated from Eq.(1)^32^, where V_m_ is molar volume of the spinel material, S is the electrolyte-accessible surface area of the electrode and (dE)/(dx) is the slope of the open-circuit potential vs. mobile ion concentration x. Because the three samples are all LiNi_0.5_Mn_1.5_O_4_, V_m_, F and (dE)/(dx) in Eq(1) are similar. The Li-ion diffusion coefficient depends on the value of S and B. With the similar value of B, the electrolyte-accessible surface area of HI-LNMO is much larger than that of SO-LNMO. So the Li-ion diffusion coefficient of HI-LNMO is lower, due to its lower Mn^3+^ content.


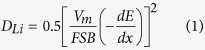


**Figure 8:**
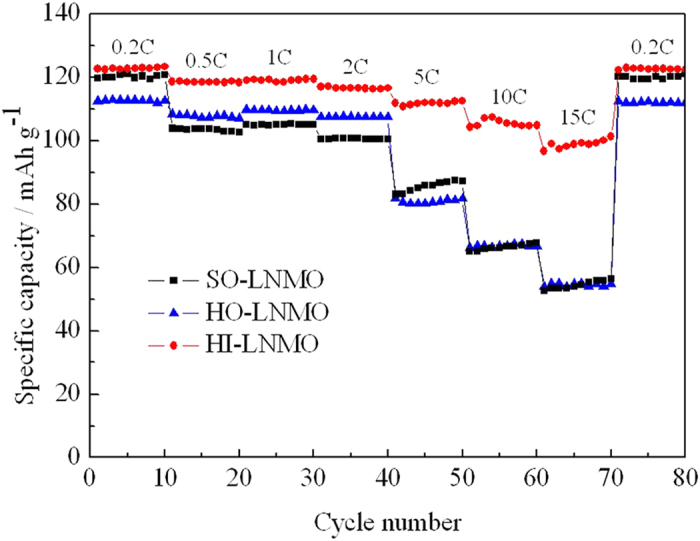
Rate capability of LiNi_0.5_Mn_1.5_O_4_ with different microstructures.

The rate capability of the LiNi_0.5_Mn_1.5_O_4_ products with different microstructures are compared and shown in Fig. 8. As can be clearly seen, HI-LNMO exhibits the best high-rate performance. HI-LNMO is still able to deliver a high specific capacity over 100 mAh g^−1^ at 15 C, equivalent to as high as 82.1% of discharge capacity at 0.2 C. However, SO-LNMO and HO-LNMO show poor high rate performance, the discharge capacity at 15 C drops to as low as 45.4% and 50.1% of that at 0.2 C, respectively. Mn^3+^ in spinel can enhance the conductivity, which is beneficial to high rate performance^11^,^12^. However, as discussed above, HI-LNMO with the lowest content of Mn^3+^ unexpectedly exhibits the best high-rate performance. Therefore, this good high-rate performance of HI-LNMO could be attributed to its hierarchical microstructure, illustrated in Figure S7, in which the submicrometer-sized primary particles obviously increase the active specific surface area for electrochemical reactions and shorten the diffusion distance for Li^+^ ions. On the other hand, it is difficult for all the primary particles to electronically contact with conducting agents uniformly. Large current will flow through the places contact with conducting agent. The connected microstructure makes every primary particle share the current and be charged/discharged efficiently. Generally, a high overpotential occurs on the particle due to the relatively high resistance which comes from a high interface resistance between the primary particles. However, in the case of hierarchical microstructure, the interface resistance between the primary particles is lower because they interconnect to each other with less interface rather than contact with interface. In other words, the good high-rate performance of HI-LNMO is partly attributed to its higher conductivity between the primary particles.

The excellent high-rate performance of HI-LNMO is further demonstrated, as shown in Figure S8, by the lowest charging plateau voltage and the highest discharging plateau voltage at the same charging/discharging rate of 5 C in cycling tests, indicating the smallest polarization.

Although rate performance can be significantly improved by preparing nanosized materials^33^,^34^, the application of nanosized materials is challenged by the following two facts: (1) they cannot be coated on the current collector as densely as micrometer-sized materials^35^, which reduces the energy density and (2) the very large specific surface area of nanosized materials cause more side reactions such as decomposition of the electrolyte and dissolution of Mn, leading to a lower durability and cycling stability^36^. Here, HI-LNMO with excellent rate capability has a BET specific surface area of as low as 2.1 m^2^ g^−1^. Compared with nano-sized materials (15.5 m^2^ g^−1^)^33^, this relatively small specific surface area is expected to promote the cycling performance and durability.

**Figure 9:**
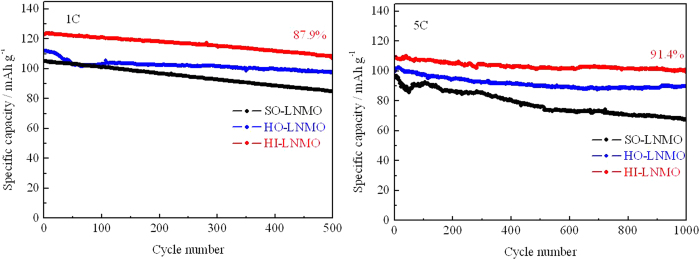
Cycling performance of LiNi_0.5_Mn_1.5_O_4_ with different microstructures rates of 1 C and 5 C.

The cycling performances at various charging/discharging rates (1 C and 5 C) of the homemade LiNi_0.5_Mn_1.5_O_4_ with different microstructures are compared, as shown in Fig. 9. After 500 cycles at 1C and 1000 cycles at 5 C, HI-LNMO shows the highest discharge capacity and the best capacity retention among the three samples. The excellent cycling performance of HI-LNMO is attributed to the following two factors: (1) a lower content of Mn^3+^ which decreases the speed of Mn dissolution and Jahn-Teller distortion and (2) the existence of space among the primary particles in the hierarchical microstructure which can buffer the volume change occurring in Li^+^ insertion/extraction process.

Interestingly, for HI-LNMO, the capacity retention after 1000 cycles at 5C is 91.4%, higher than 87.9% after 500 cycles at 1C. That is, the same sample HI-LNMO cycled at a larger current density unexpectedly shows a higher capacity retention after more cycles. This indicates that an irreversible structure change during the repeated Li^+^ insertion/extraction, which usually occurs more seriously during cycling at a higher rate, is not the dominant factor for capacity fading. It should be pointed out that it takes only 582 hours for 1000 cycles at 5C, approximately as half as 1067 hours for 500 cycles at 1C. It means that regardless of a lower current density and less cycle numbers, a longer duration of experimental process leads to a more serious capacity fading. In other words, the side reactions between the electrode and the electrolyte are the dominant factor for capacity fading. This supports that there is a trade-off between the lifetime and the high-rate performance in terms of the optimized specific surface area.

## Conclusions

LiNi_0.5_Mn_1.5_O_4_ micro-particles with solid, hollow and hierarchical microstructures were synthesized separately and compared. The following conclusions could be made. (1) It is a feasible way to control the particle microstructure of LiNi_0.5_Mn_1.5_O_4_ spinel by using manganese compounds as precursors and controlling the microstructures of the precursors. (2) An inhomogeneous migration of Ni and Mn atoms could introduce Mn^3+^ and impurity phase in the spinel product. (3) A hierarchical microstructure with the primary particles interconnected has small interface resistance. The space in the secondary particles can accommodate electrolyte and buffer volume change during cycling. Together with its appropriate specific surface area, HI-LNMO has both optimized rate capability and cycling stability. (4) Side reactions between the electrode and the electrolyte, rather than repeated Li^+^ insertion/extraction, is the dominant factor for capacity fading during cycling.

## Additional Information

**How to cite this article**: Xue, Y. *et al.* Investigation on preparation and performance of spinel LiNi_0.5_Mn_1.5_O_4_ with different microstructures for lithium-ion batteries. *Sci. Rep.* **5**, 13299; doi: 10.1038/srep13299 (2015).

## Supplementary Material

Supplementary Information
