# Supplementary material for: Investigation on preparation and performance of spinel LiNi0.5Mn1.5O4 with different microstructures for lithium-ion batteries
Source: Sci Rep. 2015 Aug 24;5:13299. doi: 10.1038/srep13299 (PMC4547101; doi:10.1038/srep13299)
Supplement: Supplementary Information [file srep13299-s1.pdf]

Supporting Information

Investigation on Preparation and Performance of Spinel $LiNi_{0.5}Mn_{1.5}O_4$ with Different Microtructures for Lithium-ion Batteries

Yuan Xue[a], Zhenbo Wang[a*], Lili Zheng[a], Fuda Yu[a], Baosheng Liu[a], Yin Zhang[a] and Ke Ke[a,b]

[a] School of Chemical Engineering and Technology, Harbin Institute of Technology, No. 92 West-Da Zhi Street, Harbin, 150001 China.

[b] Chilwee Power Co. Ltd., No. 12 Zhizhou Road, Xinxing Industrial Park, Zhicheng, Changxing, Zhejiang Province 313100, China

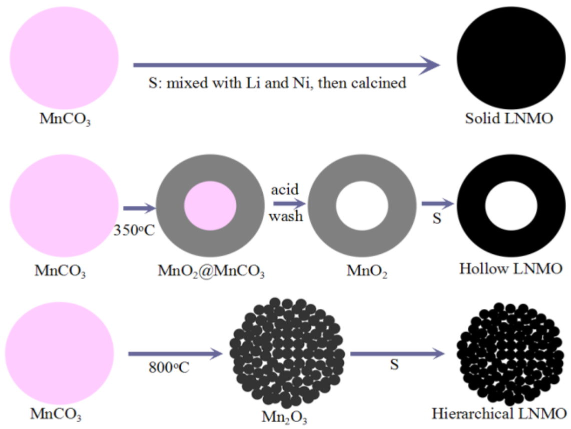

Figure S1. Schematic illustration of the controlled preparation of $LiNi_{0.5}Mn_{1.5}O_4$ with different microstructures.

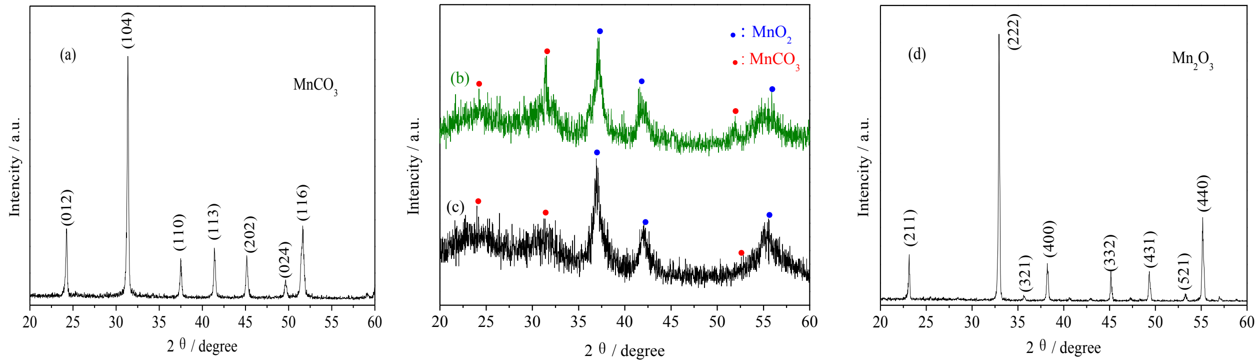

Figure S2. XRD patterns of materials obtained during the procedure of synthesizing precursors: (a) $MnCO_3$ precipitate before calcination, (b) $MnO_2/MnCO_3$ composite obtained after calcinating the precipitate at 350 °C, (c) MnO_2 obtained after acid-washing the $MnO_2/MnCO_3$ mixture, and (d) Mn_2O_3 obtained after calcinating the $MnCO_3$ precipitate at 800 °C.

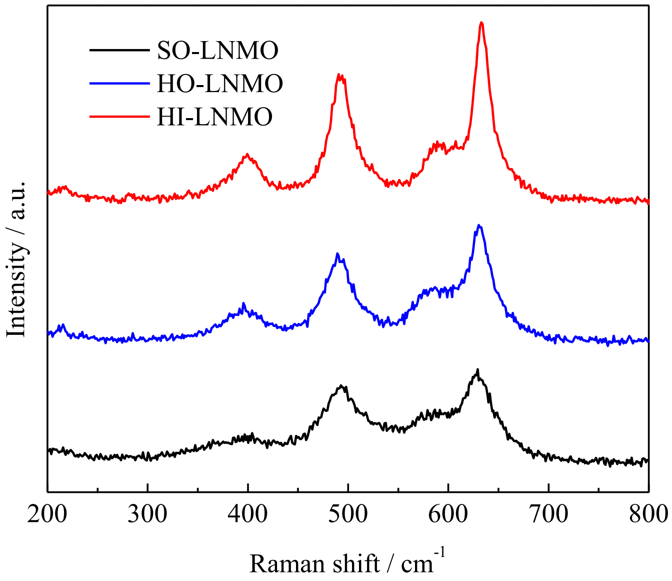

Figure S3. Raman spectra of $LiNi_{0.5}Mn_{1.5}O_4$ with different microstructures.

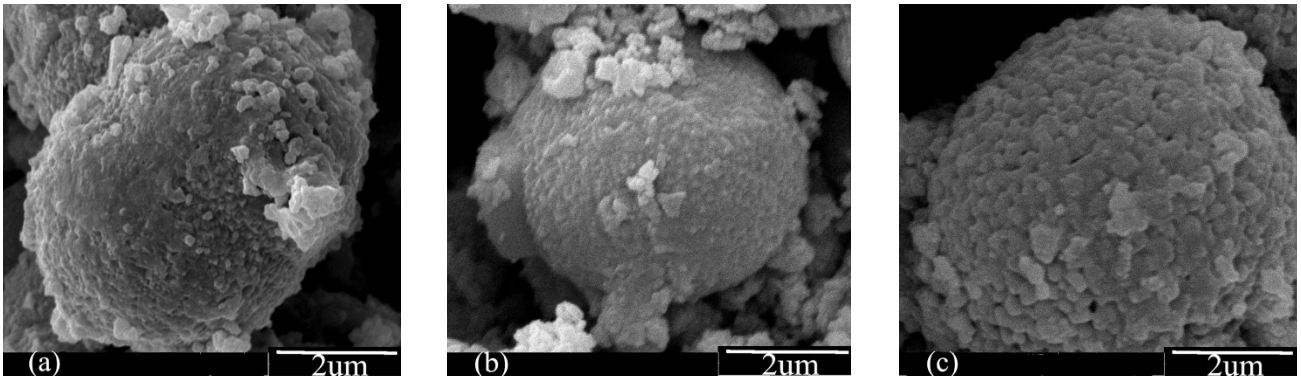

Figure S4. SEM micrographs of the mixture after mixing process of different methods. (a) SO-LNMO, (b) HO-LNMO and (c) HI-LNMO.

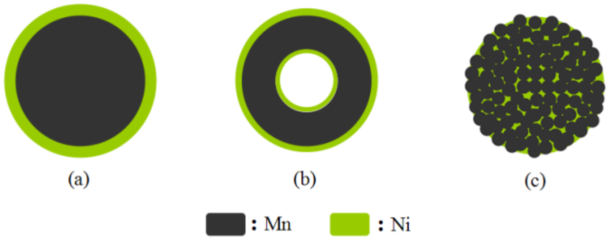

Figure S5. Schematic illustration of the distribution of Ni and Mn elements in the mixture after mixing process of different methods. (a) SO-LNMO, (b) HO-LNMO and (c) HI-LNMO.

Sample	Mn (mg L^{-1})	Ni (mg L^{-1})
SO-LNMO	20.13	6.926
HO-LNMO	19.21	6.391
HI-LNMO	18.15	6.252

Table S1. ICP results of $LiNi_{0.5}Mn_{1.5}O_4$ with different microstructures.

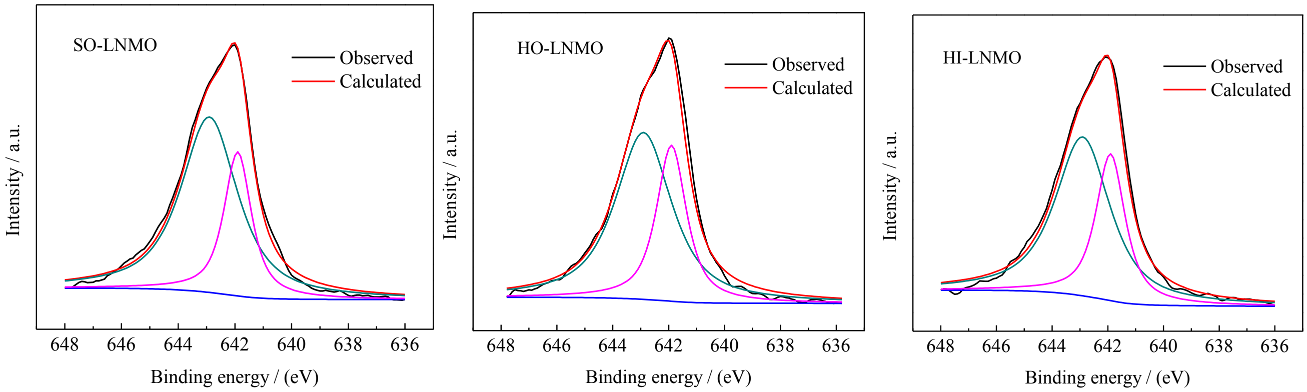

Figure S6. Mn 2p3/2 XPS spectra fitting of $LiNi_{0.5}Mn_{1.5}O_4$ with different microstructures.

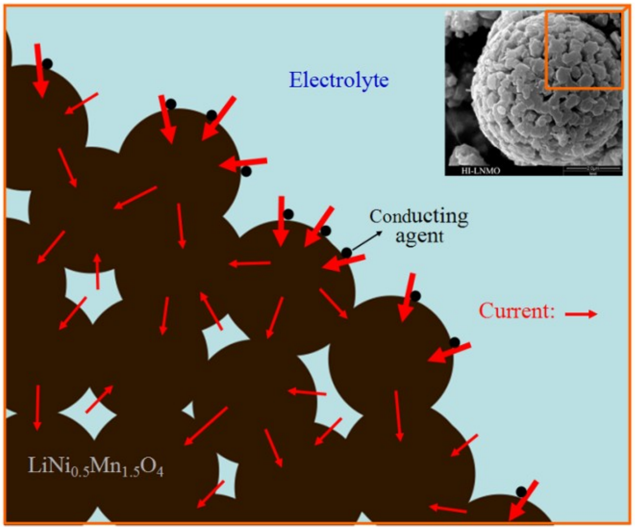

Figure S7. Schematic illustration of the submicro/micro hierarchical structure with primary particles connected.

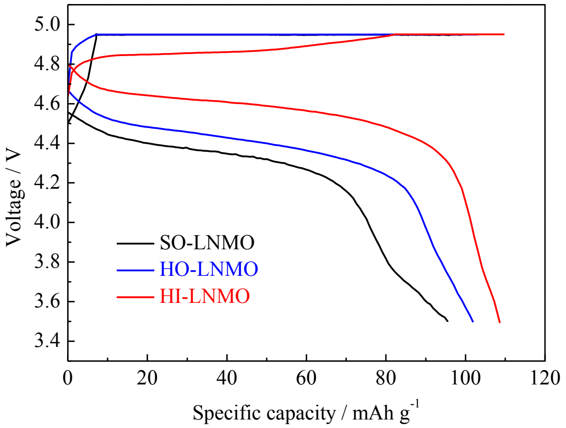

Figure S8. Charge and discharge capacity curves at 5 C of $LiNi_{0.5}Mn_{1.5}O_4$ with different microstructures.
